# Is Nighttime Really Not the Right Time for a Laparoscopic Cholecystectomy?

**DOI:** 10.1155/2018/6076948

**Published:** 2018-07-29

**Authors:** Anna C. M. Geraedts, Meindert N. Sosef, Jan Willem M. Greve, Mechteld C. de Jong

**Affiliations:** Department of Surgery, Zuyderland Medical Centre, Heerlen & Sittard, Netherlands

## Abstract

**Purpose:**

The impact of an out-of-hours laparoscopic cholecystectomy on outcome is controversial. We sought to determine the association between an out-of-hours procedure and postoperative complications within 90 days.

**Methods:**

Between 2014 and 2016, 1553 laparoscopic cholecystectomies were performed. Therapeutic, operative, and outcome data were prospectively collected and analyzed. We defined out of hours as during weekends, national holidays, and daily between 5PM and 8AM.

**Results:**

Most patients operated on were female (n=988; 63.6%) and the majority of procedures were electives (n=1341; 86.3%). Although all procedures were performed with a laparoscopic intent, 42 (2.7%) were converted to open procedure. In total, 145 (9.3%) procedures were out of hours, all nonelective, and in most cases for acute cholecystitis (n=111; 7.1%). Overall, there were 212 complications in 191 patients (12.3%), most (n=153; 9.9%) classified as minor. The conversion rate in the out-of-hours group was significantly higher (9.7% vs 2.0%; p<0.001). While univariate analyses revealed out-of-hours procedure (OR=1.83; p=0.008) to be associated with an increased risk of complications, when controlling for confounding factors by multivariate analysis, this association was not found. However, operation by surgical staff (OR=1.71) and conversion to laparotomy (OR=3.74) were found to be independently associated with an increased risk of complications (both* p*<0.05), while an emergency procedure tended to be associated with postoperative morbidity (OR=1.82*; p*=0.069).

**Conclusion:**

An out-of-hours laparoscopic cholecystectomy was not found to be an independent risk factor for developing postoperative morbidity and time of day should therefore only be a relative contraindication.

## 1. Introduction

Laparoscopic cholecystectomy is currently the gold standard procedure for gallbladder removal and is presently the most commonly performed abdominal intervention in the Western World [1]. The first cholecystectomy was performed by Langenbuch in 1882, while the first laparoscopic cholecystectomy was described by Mühe in 1985 [2]. Two years later, the first acknowledged laparoscopic cholecystectomy by means of four trocars was performed by Mouret [3]. However, with a wide variety of techniques to perform this laparoscopic cholecystectomy, it was initially a procedure with a high risk for complications. Especially injuries to the biliary duct remained a serious drawback. Most of these injuries arise from misidentifications, with either the common bile duct or an aberrant bile duct as the cystic duct. In order to prevent such injuries, Strasberg proposed the so-called critical view technique [4].

While most laparoscopic cholecystectomies are in an elective setting and therefore planned during daytime, at times it is necessary to perform these procedures out of hours. The circumstances under which these patients are operated on differ from those during office hours. Specifically, indications for surgery are generally different (emergency only); only the surgeon and resident on call are available for the operation and hospital resources are more limited.

For other procedures (e.g., hip surgery or cardiac surgery), ambivalent data regarding the safety of performance of these procedures during nighttime have been published, with some authors stating that the risk of complications is increased during out-of-hours procedures [5–7]. For laparoscopic cholecystectomy, the impact on outcome of an out-of-hours procedure is unclear. Therefore, the primary aim of this study was to determine the association between an out-of-hours laparoscopic cholecystectomy and postoperative complications within 90 days compared to daytime procedures. Our hypothesis was that out-of-hours laparoscopic cholecystectomy would result in an increased rate of complications.

## 2. Methods

Between 1^st^ January 2014 and 31^st^ December 2016, 1553 patients underwent a laparoscopic cholecystectomy at Zuyderland Medical Center, Heerlen and Sittard, the Netherlands. All data were prospectively collected in a database and retrospectively analyzed. Approval from our Institutional Review Board for this study was obtained. The primary outcome was all-cause morbidity. This was a composite outcome and included any documented complication within 90 days of surgery.

The preferred method for the removal of the gall bladder in our center—be it elective or in an emergency setting—is via laparoscopy. For this laparoscopic cholecystectomy we follow the critical view technique of safety proposed by Strasberg [8]; achievement of this critical view of safety is mentioned in the operative report.

Patients are usually referred to our outpatient department by their general practitioner with symptomatic gallbladder stones and subsequently planned for an elective laparoscopic cholecystectomy. Other indications for surgery include referral from our gastroenterologists because of a biliary pancreatitis or bile duct stones. When patients present with an acute cholecystitis they are preferably operated on in an acute setting or sometimes electively planned for a laparoscopic cholecystectomy à froid based on patient characteristics, durations of symptoms in accordance with the current national guidelines [9]. For the purpose of the current study, we defined out of hours as during weekends, national holidays, and daily between 5PM and 8AM.

### 2.1. Data Collection

Due to the retrospective nature of the study, it was not deemed possible to calculate a number needed to treat or to perform a power-analysis. Standard demographic and clinical data were collected on each patient such as age, gender, and indication for surgery. Furthermore, it was noted if patients had undergone an endoscopic retrograde cholangiopancreatography (ERCP) before surgery. Operative details were also mentioned, including the use of antibiotics, whether the critical view of safety was achieved [4], the occurrence of bile spill, conversion to laparotomy, operative time, and operative blood loss. Perioperative morbidity and mortality were noted, defined as during the same hospitalization or within 90 days of surgery. Furthermore, complications were scored according to the Clavien grading system [10].

### 2.2. Statistical Analyses

Summary statistics were obtained and presented as percentages or median values. Upon comparing categorical data, the *χ*2-test, or if deemed appropriate Fisher's exact test, was used, while the Mann–Whitney* U* test was used to compare continuous data. Factors associated with perioperative morbidity were examined using univariate and multivariate regression analyses. The odds ratios and the 95% confidence intervals (CI) were estimated and a* p* value of less than 0.05 was considered significant. All statistical analyses were performed using IBM SPSS Statistics for Macintosh, Version 25.0 (IBM Corp. IMB SPSS statistics, Armonk, NY).

## 3. Results

### 3.1. Patient and Operative Characteristics

Between 1^st^ January 2014 and 31^st^ December 2016, 1553 patients underwent an intended laparoscopic cholecystectomy at our institution and these were queried from our database. The characteristics of these patients are detailed in Table 1. Most patients were female (*n*=988; 63.6%), with a median age of 55 [range: 14-90]. The indications for undergoing a laparoscopic cholecystectomy are further specified in Table 1. The timing of surgery was elective in the majority of patients (*n*=1341; 86.3%), while 212 patients (13.7%) underwent emergency surgery. Specifically, nine of these patients (0.6%) were planned to undergo an elective procedure but had to undergo an acute cholecystectomy prior to this planned date.

Overall, the median operative time was 60 minutes [12-360], with a median estimated blood loss of 10 mL [0-1400].

While all patients were intended to undergo a laparoscopic cholecystectomy, conversion to an open procedure occurred in 42 cases (2.7%). Reasons for conversion included, among others, hostile abdomen (*n*=13; 0.84%); perforation of the gallbladder (*n*=11; 0.71%); insufficient overview of the hilar structures (*n*=1; 0.71%) and suspected injury of the common bile duct (*n*=2; 0.2%).

### 3.2. Out-of-Hours Procedures

145 patients (9.3%) underwent an out-of-hours procedure. Notably, all of these procedures were nonelective (*n*=145; 9.3%) and in most cases the indication for surgery was an acute cholecystitis (*n*=111; 7.1%).  Table 2 further specifies the details of patients who underwent an out-of-hours procedure. Importantly, upon comparing the characteristics of these two groups, the included patients were very heterogeneous, as major base-line differences were observed.

The operative time was significantly longer during out-of-hours procedures (70 minutes* vs *54 minutes;* p*=0.033); and these procedures were also associated with a higher median estimated blood loss (30 mL* vs *10 mL;* p*<0.001). Furthermore, the median age of patients was higher (60* vs* 55;* p*=0.012), bile spill occurred more often (42.8%* vs* 33.5%;* p*=0.027), and the critical view of safety was mentioned in less operative notes (86.2%* vs* 94.5%;* p*=0.020) during an out-of-hours procedure. The conversion rate in the out-of-hours group was also significantly higher (9.7%* vs* 2.0%;* p*<0.001).

### 3.3. Emergency Procedures

To gain more insight into the possible differences between patients operated on during daytime and after-hours, we explored the subgroup of patients undergoing a laparoscopic cholecystectomy as an emergency case. These results are displayed in Table 3. Interestingly, upon comparing patients who underwent their emergency procedure during daytime to those whose procedure was performed at night, there were no differences regarding gender, age, previous abdominal surgery, or indication for surgery (all* p*>0.05). Furthermore, there were no differences regarding bile spill, operative time, nor estimated blood loss (all* p*>0.05). The only difference during the procedure was the seniority of the surgeon performing the operation, as emergency cases during business hours were more often performed by a surgical resident rather than by a surgical staff member compared with out-of-hours procedures (*p*<0.001).

### 3.4. Postoperative Morbidity

Overall, the complication rate during hospital admittance after an intended laparoscopic cholecystectomy, or during the 90-day postoperative period, was 12.3% (*n*=191). While most (*n*=153; 9.9%) complications were classified as minor complications (Clavien <3),(10) 38 patients (2.4%) experienced a major complication following cholecystectomy. The specifics of postoperative morbidity are detailed in Table 4.

Importantly, five patients (0.3%) died within 90 days. Notably, three of these patients succumbed after undergoing their elective, daytime laparoscopic cholecystectomy for symptomatic gallstone disease. Two patients were operated on in an emergency setting.

There were notable differences in the incidence of complications between patients who underwent, and elective laparoscopic cholecystectomy compared with an emergency procedure (*n*=151 (11.3%)* vs n*=40 (19.0%);* p*=0.002). Moreover, the incidence of postoperative morbidity was also different between patients where bile was spilled compared with no spillage (*n*=443 (33.1%)* vs n*=91 (43.3%);* p*=0.004). Furthermore, the incidence of complications was higher during out-of-hours procedures (*n*=28 (19.3%)* vs n*=163 (11.6%);* p*=0.007). Additionally, the occurrence of more severe postoperative morbidity bordered on being a significantly higher among patients who underwent an out-of-hours laparoscopic cholecystectomy (major complications (Clavien ≥3):* n*=7 (4.8%)* vs n*=31 (2.2%);* p*=0.052) [10].

When further exploring the subgroup of patients who underwent an emergency procedure, the incidence of complications was comparable between patients who were operated on during daytime (*n*=7; 4.8%) and those operated on out of hours (*n*=31; 2.2%) (*p*=0.8461). Moreover, the risk of developing postoperative morbidity was also comparable for emergency laparoscopic cholecystectomies during nighttime and daytime (OR=1.08 [95%-CI 0.51-2.78];* p*=0.85).

When solely addressing the subgroup of patients who underwent a laparoscopic cholecystectomy for acute cholecystitis (*n*=195), 111 patients (56.9%) were operated on out of hours, while 84 patients (43.1%) were operated on during business hours. Overall, 37 patients (19.0%) within this subgroup developed postoperative morbidity. In over three-quarters of patients who developed complications, these were minor complications (Clavien <3) (*n*=29; 78.4%). Upon comparing patients who were operated on out of hours* versus* during business hours, no difference in the overall complication rate was observed (*n*=21 (18.9%)* versus n*=16 (19.1%) (*p*=0.99)). Moreover, when dividing the complications into minor (Clavien <3) and major (Clavien ≥3), the same pattern was observed. Specifically, minor complications occurred in 16 patients (14.4%) who were operated on out of hours compared with 15 patients (17.9%) who were operated on during business hours (*p* =0.31). For major complications, there was a similar trend: five patients (4.5%) who were operated on out of hours developed minor morbidity, compared with one patient (1.2%) during business hours (*p* =0.40).

Upon examining the risk factors for complications following a laparoscopic cholecystectomy (Table 5), univariate analyses revealed multiple factors thought to be associated with an increased risk. Specifically, an out-of-hours procedure (OR=1.83;* p=*0.008); a nonelective procedure (OR=1.58;* p*=0.018); an operation by a surgical staff member (OR=1.92;* p*=<0.001) and conversion to a laparotomy (OR=4.70;* p*<0.001) were found to be associated with an increased risk of complications. However, after controlling for possible confounding factors by performance of a multivariate analysis, the only factors found to put patients at an increased risk for development of complications were an operation by a surgical staff member (OR=1.71;* p*=0.001) and conversion to a laparotomy (OR=3.74;* p*<0.001). Moreover, an emergency procedure tended to be associated with postoperative morbidity (OR=1.82;* p*=0.069).

## 4. Discussion

While a laparoscopic cholecystectomy is generally performed as an elective procedure, some patients undergo this operation in an emergency setting, frequently out of hours. With the current study, we aimed to determine if patients who underwent such an out-of-hours laparoscopic cholecystectomy were at an increased risk of developing complications.

We found that, in our series, a considerable number of patients developed complications after a laparoscopic cholecystectomy (12.3%), although these rates are comparable to those reported by others [11–13]. The incidence of complications was higher during out-of-hours procedures (19.3%* vs *11.6%), whereas previous studies on laparoscopic cholecystectomies during nighttime did not show any significant differences in the rate of complications [11, 14]. Moreover, upon comparing daytime and nighttime laparoscopic cholecystectomies, we found that, for out-of-hours procedures, the conversion rate was significantly higher (9.7%* vs* 2.0%), the estimated blood loss was significantly increased (30 mL* vs* 10 mL), and the operative time bordered on being significantly longer (70 min* vs* 54 min). We reason that these factors could all be viewed as indicators for a more technically challenging procedure.

On another note, there was an ongoing increase of centralization of care with a concomitant rise in dedicated surgical teams [15–18]. While data have shown an improvement in outcomes due to these changes in patterns of care [19–21], there was a potential drawback of this centralization for noncentralized procedures. Due to the performance of large, elective surgeries during daytime, the performance of emergency surgery during nighttime remains common. Moreover, laparoscopic cholecystectomy is viewed as general surgery at our facility, meaning that these procedures do not have to be performed by a specialized gastrointestinal surgeon. Especially at nighttime, it was at the discretion of the surgeon on call whether to perform the procedure, postpone until the next day, or consult the gastrointestinal surgeon on call. Therefore, it was not only important to establish the outcomes of these surgeries during out of hours but also to identify potential risk factors for complications and to select possible high-risk patients.

Although on univariate analyses, multiple risk factors for development of postoperative morbidity were identified within our cohort, only the association between a higher risk of complications and performance of the procedure by surgical staff (OR=1.71;* p*=0.001) or after conversion (OR=3.74;* p*<0.001) was confirmed after correcting for possible confounders. Furthermore, an emergency procedure bordered on being associated with postoperative morbidity (OR=1.82;* p*=0.069). The variables adjusted for are time of day, timing of surgery, the level of the performing surgeon, and conversion to a laparotomy, as depicted in Table 5. Our hypothesis is that the factors found to be associated with outcome on univariate analysis are merely surrogates for the most important risk factor for the occurrence of postoperative morbidity: a more complex procedure, which on its turn was predominantly caused by the severity of the disease. Specifically, the association between an increased risk of complications and the performance of the operation by surgical staff was likely also based on selection bias; i.e., the more complicated the surgery, the more likely this was performed by surgical staff other than a resident. Interestingly, while performance of an out-of-hours procedure seemed to be associated with an increased risk of postoperative morbidity (OR=1.83;* p*=0.008), this association was not confirmed after controlling for possible confounding factors (OR=0.74;* p*=0.43). It is therefore possible that the result for this variable on univariate analysis merely depicts the influence of other factors on the occurrence of complications (e.g., all procedures performed out of hours were acute, the ratio of conversion was higher in this group, and the majority of procedures were performed by surgical staff) and not of the actual time of the procedure. To date, no validated risk stratification algorithm exists to accurately predict severity of the disease at time of surgery [22]. Especially, there are no preoperative factors known to be sensitive and specific enough for clinicians to truly rely on, to predict a difficult procedure.

Timing of surgery is one of the factors described in literature, to influence the risk of complications due to its effect on the case complexity [23–27]. The evidence to support early laparoscopic cholecystectomy (i.e., less than 24 hours after diagnosis) for biliary colic is only based on one trial and this is therefore not deemed standard practice [23, 24]. The optimal timing in case of an acute cholecystitis also remains controversial. Whereas a recent review [25] comparing early (less than seven days of clinical presentation) versus delayed laparoscopic cholecystectomy (more than six weeks after index admission) found no difference in bile duct injury or other serious complications, Banz et al. [26] found an overall complication rate of 6%, which increased significantly with delaying the procedure (from 5.7% to 13% after 6 or more days of cholecystitis) in their population-based-analysis. In concordance with our national guidelines, this supports performance of a laparoscopic cholecystectomy within the first 5 days of presentation rather than delaying for six weeks, i.e., performing laparoscopic cholecystectomy à froid [9]. In addition, there seems to be no evidence to support operating within the first 24 hours of presentation [27]; therefore these data do not necessitate performance of an out-of-hours laparoscopic cholecystectomy for biliary colic or for acute cholecystitis. Interestingly, Siada et al. [28] found that costs did not differ between the day and night groups for acute cholecystitis.

Additional risk factors for the development of complications following laparoscopic procedures have been identified in literature. Elderly patients have an increased risk of complications, due to worsening of their already fragile general condition caused by a systemic infection. Furthermore, patients with extensive cardiopulmonary comorbidity are at high risk due to possible worsening by the pneumoperitoneum during a laparoscopic cholecystectomy [29]. Lastly, patients who had extensive previous abdominal surgery may have an increased risk of perforation of bowel or large vessels due to adhesions [29]. Ultimately, all these factors should be taken into consideration when deciding whether or not to perform an out-of-hours procedure.

While our current study did not identify performance of a laparoscopic cholecystectomy after office hours as an independent risk factor for complications, we do consider limiting performances of out-of-hours surgery as the best standard of care. Moreover, while our hospital is also a trauma center, demise in the operating rooms during nighttime should be reduced to a minimum, due to the ever-present potential for arrival of trauma victims. In order to achieve this, we firstly advise facilitating an operating room and surgical team dedicated to all emergency surgery during daytime so as to minimize the number of out-of-hours procedures in general. Moreover, by creating more operative time to perform laparoscopic cholecystectomies in an elective setting, the waiting lists will be shortened, resulting in a possibly lower incidence of emergency laparoscopic cholecystectomies and therefore less out-of-hours procedures. Moreover, as out-of-hours procedures are possibly more difficult procedures in proven, sicker patients, it could even be contemplated to have these procedures performed solely by the most technically advanced surgeon, i.e., surgical staff rather than a resident.

Finally, we think that patients who are thought to have an increased risk for complications (e.g., possibly more advanced disease, elderly patients, or patients with large comorbidity) should be selected for priority daytime surgery, when more resources are available.

The current study has several limitations. Like all retrospective studies, selection bias may have influenced the choice patients operated on during night time. This in turn could have biased our risk factor analysis. Furthermore, exposure or outcome assessment could not be controlled as we had to rely on the accuracy of the recordkeeping. Moreover, due to the small number of patients in the subgroup of acute cholecystitis and especially the relatively low incidence of particularly major complications, no further statistical inferences could be conducted for patients who underwent a laparoscopic cholecystectomy for acute cholecystitis. Additionally, due to the unavailability of necessary data, further inferences within this subgroup were limited.

In conclusion, performance of an out-of-hours laparoscopic cholecystectomy was not found to be an independent risk factor for developing postoperative morbidity and time of day should therefore only be viewed as a relative contraindication. However, the risks of performance of an out-of-hours procedure should be weighed against its benefits and therefore select high-risk patients (i.e., possible complex cases) should be prioritized to undergo a laparoscopic cholecystectomy during the next day, instead of during nighttime.

## 5. Conclusion

An out-of-hours laparoscopic cholecystectomy was not found to be an independent risk factor for developing postoperative morbidity and time of day should therefore only be a relative contraindication.

## Figures and Tables

**Table 1:** Patient clinicopathologic and operative characteristics.

**Variable**	**Number (**%**); *n*=1553**
*Patient characteristics*	

Gender (female)	988 (63.6)
Age (median [range])	55 [14-90]
Previous abdominal surgery	
None	1177 (75.8)
Laparoscopic	219 (14.1)
Laparotomy	157 (10.1)

*Preoperative details*	

Indication for surgery–*multiple options possible*	
Bile stone colics	1055 (67.9)
Cholecystitis à froid	138 (8.9)
Acute cholecystitis	195 (12.6)
Bile duct stones	59 (3.8)
Biliary pancreatitis	103 (6.6)
Other	42 (2.7)
Timing of surgery	
Elective	1341 (86.3)
Emergency	212 (13.7)
Out-of-hours procedure	145 (9.3)

*Perioperative variables*	

Usage of antibiotics	
No	971 (62.5)
Yes–prior to operation	400 (25.7)
Yes–after bile spill	181 (11.7)
Surgeon	
Surgical resident	972 (62.6)
Surgical staff	581 (37.4)
Critical View of Safety mentioned	1455 (93.7)
Bile spill	534 (34.4)
Conversion to laparotomy	42 (2.7)
Operative Time (median [range])	60 [12-360]
Estimated Blood Loss (median [range])	10 [0-1400]

**Table 2:** Patient clinicopathologic and operative characteristics; comparing out-of-hours procedures to those during business hours.

**Variable**	**Number (**%**)**		
	**Out-of-hours (*n*=145)**	**During Business Hours (*n*=1408)**	***p*-value**
*Patient characteristics*			

Gender (female)	85 (58.6)	903 (64.1)	0.19
Age (median [range])	60 [19-89]	55 [14-90]	0.012
Previous abdominal surgery			
None	111 (76.5)	1064 (75.6)	0.22
Laparoscopic	15 (10.3)	204 (14.5)	
Laparotomy	19 (13.1)	138 (9.8)	

*Preoperative details*			

Indication for surgery			
Bile stone colics	23 (15.9)	1032 (73.3)	<0.001
Acute cholecystitis	111 (76.6)	84 (6.0)	
Other	11 (7.6)	315 (22.4)	
Timing of surgery			
Elective	0 (0)	1342 (95.3)	<0.001
Emergency	145 (100)	67 (4.7)	

*Perioperative variables*			

Usage of antibiotics			
No	36 (24.8)	935 (66.4)	<0.001
Yes–prior to operation	101 (69.7)	299 (21.2)	
Yes–after bile spill	8 (5.5)	173 (12.3)	
Surgeon			
Surgical resident	33 (22.8)	938 (66.6)	<0.001
Surgical staff	112 (77.2)	469 (33.3)	
Critical View of Safety mentioned	125 (86.2)	1330 (94.5)	0.020
Bile spill	62 (42.8)	472 (33.5)	0.027
Conversion to laparotomy	14 (9.7)	28 (2.0)	<0.001
Operative Time (median [range])	70 [16-206]	54 [12-360]	0.033
Estimated Blood Loss (median [range])	30 [0-1000]	10 [0-1400]	<0.001

**Table 3:** Patient clinicopathologic and operative characteristics; comparing emergency in daytime to those at night during business hours.

**Variable**	**Number (**%**)**		
	**Out-of-hours (*n*=145)**	**During Business Hours (*n*=67)**	***p*-value**
*Patient characteristics*			

Gender (female)	85 (58.6)	33 (49.3)	0.242
Age (median [range])	60 [19-89]	59 [20-85]	0.758
Previous abdominal surgery			
None	111 (76.6)	54 (80.6)	0.320
Laparoscopic	15 (10.3)	9 (13.4)	
Laparotomy	19 (13.1)	4 (6.0)	

*Preoperative details*			

Indication for surgery			
Bile stone colics	23 (15.9)	5 (7.5)	0.527
Acute cholecystitis	111 (76.6)	53 (79.1)	
Other	11 (7.6)	9 (13.4)	

*Perioperative variables*			

Usage of antibiotics			
No	36 (24.9)	17 (25.4)	0.818
Yes–prior to operation	101 (69.7)	45 (67.2)	
Yes–after bile spill	8 (5.5)	5 (7.5)	
Surgeon			
Surgical resident	33 (22.8)	42 (62.7)	<0.001
Surgical staff	112 (77.2)	25 (37.3)	
Critical View of Safety mentioned	125 (86.2)	61 (91.0)	0.433
Bile spill	62 (42.8)	29 (43.3)	0.802
Conversion to laparotomy	14 (9.7)	2 (3.0)	0.097
Operative Time (median [range])	60 [16-206]	70 [25-160]	0.074
Estimated Blood Loss (median [range])	30 [0-1000]	20 [0-1000]	0.817

**Table 4:** Surgical complications and mortality.

**Variable**	**Number (**%**)**
Postoperative morbidity/mortality	191 (12.3)
Type of complication–*multiple options possible*	
Surgical site infection	45 (2.9)
Bile leak / biloma	16 (1.0)
Retained stone	17 (1.1)
Abscess	19 (1.2)
Bleeding	1 (0.06)
Other	104 (6.7)
Pancreatitis	5 (0.3)
Death	5 (0.3)
Grade of complications	
Minor (Clavien <3)	153 (9.9)
Major (Clavien ≥3)	38 (2.4)

**Table 5:** Uni- and multivariate analyses assessing the association between different factors and postoperative morbidity after laparoscopic cholecystectomy.

**Prognostic Factor**	**Univariate**		**Multivariate**	
	**OR [95**%**-CI]**	***p*-value**	**OR [95**%**-CI]**	***p*-value**
Gender, male	1.07 [0.78-1.46]	0.69		
Age	1.01 [0.99-1.02]	0.10		
Out-of-hours	1.83 [1.17-2.85]	0.008	0.74 [0.35-1.57]	0.43
Timing, acute	1.59 [1.09-2.33]	0.018	1.82 [0.95-3.45]	0.069
Prior abdominal surgery	1.24 [0.88-1.75]	0.40		
Operation by surgical staff	1.92 [1.41-2.60]	<0.001	1.71 [1.24-2.36]	0.001
Bile spill	1.07 [0.78-1.47]	0.68		
Conversion to laparotomy	4.70 [2.47-8.92]	<0.001	3.74 [1.93-7.24]	<0.001

## Data Availability

Data underlying the findings of the study can be accessed by contacting the corresponding author.
